# Supplementary material for: Association between oral microbiome and seven types of cancers in East Asian population: a two-sample Mendelian randomization analysis
Source: Front Mol Biosci. 2023 Nov 21;10:1327893. doi: 10.3389/fmolb.2023.1327893 (PMC10702768; doi:10.3389/fmolb.2023.1327893)
Supplement: Supplementary file 1 [file DataSheet1.ZIP › Supplementary Materials/MR plots for tongue/tongue═╝/Prostate cancer/pheno.253_to_prostate cancer_leave_one_out.pdf]

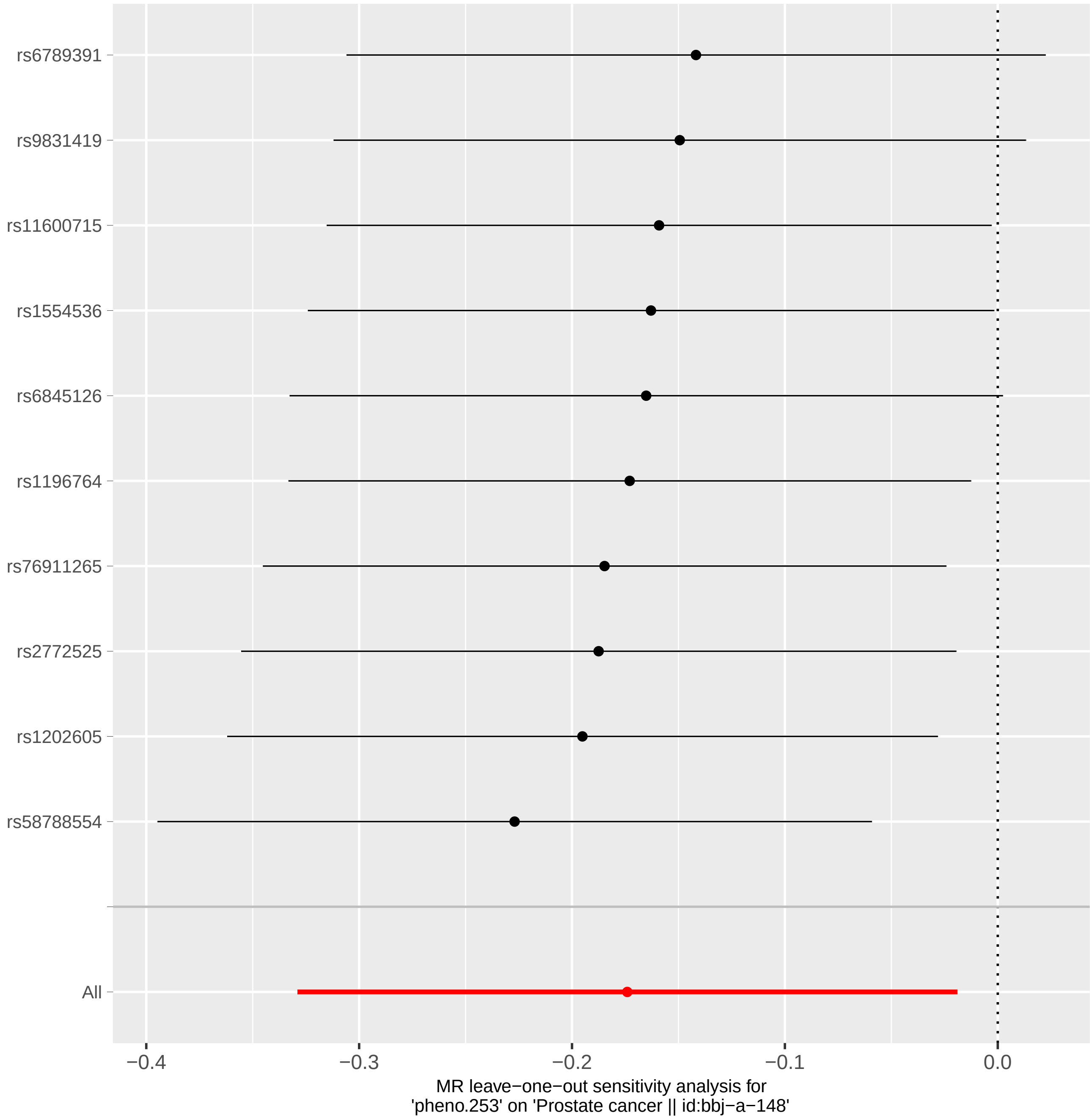

rs6789391
rs9831419
rs11600715
rs1554536
rs6845126
rs1196764
rs76911265
rs2772525
rs1202605
rs58788554
All
−0.4
−0.3
−0.2
−0.1
0.0
MR leave−one−out sensitivity analysis for
'pheno.253' on 'Prostate cancer || id:bbj−a−148'
